# Cross-Linked and Surface-Modified Cellulose Acetate as a Cover Layer for Paper-Based Electrochromic Devices

**DOI:** 10.1021/acsapm.0c01252

**Published:** 2021-03-16

**Authors:** Joice Jaqueline Kaschuk, Maryam Borghei, Katariina Solin, Anurodh Tripathi, Alexey Khakalo, Fábio A. S. Leite, Aida Branco, Miriam C. Amores de Sousa, Elisabete Frollini, Orlando J. Rojas

**Affiliations:** † Macromolecular Materials and Lignocellulosic Fibers Group, Center for Research on Science and Technology of BioResources, Institute of Chemistry of São Carlos, University of São Paulo, CP 780, 13560-970 São Carlos, São Paulo, Brazil; ‡ Department of Bioproducts and Biosystems, School of Chemical Engineering, Aalto University, Vuorimiehentie 1, FI-00076 Espoo, Finland; § Department of Chemical and Biomolecular Engineering, North Carolina State University, 27695 Raleigh, North Carolina, United States; ∥ VTT Technical Research Centre of Finland Ltd, P.O. Box 1000, FI-02044, VTT Espoo, Finland; ⊥ Ynvisible GmbH, Engesserstr. 4A 79108 Freiburg, Germany; # Ynvisible SA, Rua Quinta do Bom Retiro 12C, 2820-690 Charneca da Caparica, Portugal; ¶ Bioproducts Institute, Department of Chemical and Biological Engineering, Department of Chemistry and Department of Wood Science, The University of British Columbia, 2360 East Mall, BC V6T 1Z3 Vancouver, Canada

**Keywords:** cellulose acetate, cross-linking, hydrophobization, barrier properties, electrochromic displays

## Abstract

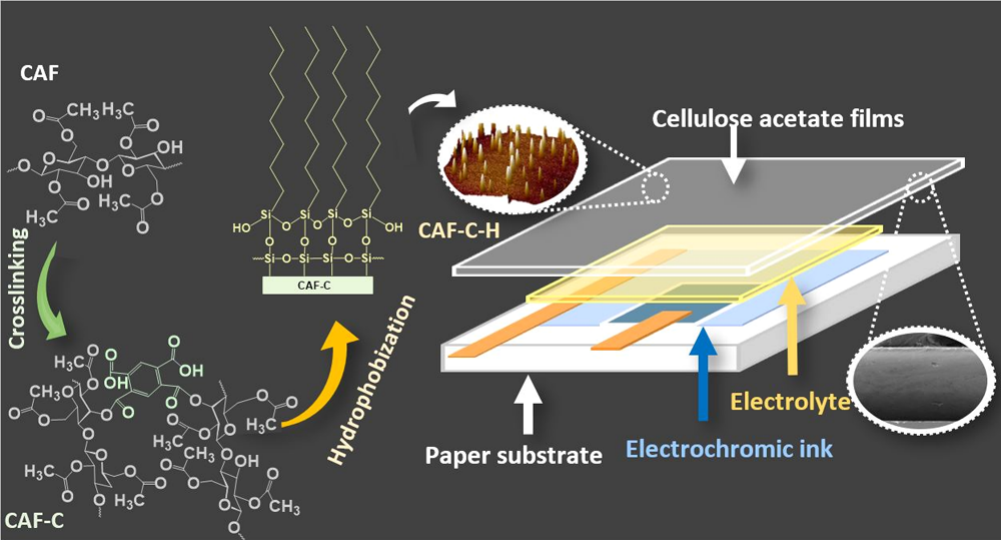

We studied the surface and microstructure of cellulose acetate (CA) films to tailor their barrier and mechanical properties for application in electrochromic devices (ECDs). Cross-linking of CA was carried out with pyromellitic dianhydride to enhance the properties relative to unmodified CA: solvent resistance (by 43% in acetone and 37% in DMSO), strength (by 91% for tensile at break), and barrier (by 65% to oxygen and 92% to water vapor). Surface modification via tetraethyl orthosilicate and octyltrichlorosilane endowed the films with hydrophobicity, stiffness, and further enhanced solvent resistance. A detailed comparison of structural, chemical, surface, and thermal properties was performed by using X-ray diffraction, dynamic mechanical analyses, Fourier-transform infrared spectroscopy, and atomic force microscopy. Coplanar ECDs were synthesized by incorporating a hydrogel electrolyte comprising TEMPO-oxidized cellulose nanofibrils and an ionic liquid. When applied as the top layer in the ECDs, cross-linked and hydrophobized CA films extended the functionality of the assembled displays. The results indicate excellent prospects for CA films in achieving environmental-friendly ECDs that can replace poly(ethylene terephthalate)-based counterparts.

## Introduction

The current environmental pressure, exacerbated by the utilization of fossil-based resources, has led to increased interest in bio-based materials.^1,2^ Those prepared from cellulose derivatives are of particular interest, given their relatively low cost and facile processing compared to unmodified cellulose, coupled with excellent physicomechanical properties.^3,4^ The most studied cellulose derivative, cellulose acetate (CA),^5^ is obtained from either heterogeneous^6−8^ or homogeneous^9^ reactions. Materials based on CA are attractive because of their solubility in a broad range of solvents, high transparency, relatively low cost, nontoxicity, biodegradability, and biocompatibility.^10^ Properties of CA vary significantly with the degree of substitution of hydroxyl groups.^11^ CAs of a low degree of substitution (<2.5) are hydrophobic but still exhibit strong intermolecular hydrogen bonding. The combination of these two characteristics favors homogenous CA films (CAF) with a competitive mechanical strength. As such, CAs are widely used in coatings, cigarette filters, textile fibers, filtration membranes, and in a plethora of medical and pharmaceutical products.^12,13^ Besides, CA can be used as a component in scaffolds and devices used in biomedical detection and imaging,^14^ energy storage,^11^ solar cells,^15^ and sensing with recyclable optical fibers.^16^

Despite the above-mentioned benefits, the inherent characteristics of CAFs still prevent their use when barrier and mechanical properties are demanded. In this work, the mechanical and barrier properties of CAFs, as well as their resistance to polar solvents were significantly improved by our developed cross-linking method using pyromellitic dianhydride (PMDA).^17,18^ Further surface modification was performed using tetraethyl orthosilicate (TEOS) and octyltrichlorosilane (OCTS), which chemically reacted with the residual hydroxyl groups, creating a hydrophobic surface rich in polysiloxane structures.^19^

The modified CAFs were applied as the top layer in a paper-based electrochromic display. Typically, electrochromic devices (ECDs) change the color under positive voltage and as a result of electrochemical redox reactions.^20,21^ The performance of ECD is influenced by the electrode substrates, electrolyte, and charge-balancing electroactive species.^22^ The main requirements for the top layer in ECDs include electrolyte stability, transparency, and the ability to hold and extend the durability of the electrochromic ink. Commonly, glass or plastic substrates such as poly(ethylene terephthalate) (PET) or poly(ethylene naphthalate) (PEN) are used in ECDs.^23^ Paper-based substrates have received increasing attention given their environmental friendliness and biodegradability.^23^ Here, we introduce ECD as a more sustainable system involving greener alternatives. This is achieved by considering the following highlights: (1) A commercial paper (PowerCoat for printed electronics) was used here as the substrate in ECD. (2) The ECD was built in a coplanar architecture where both electrodes were printed on the paper substrate. (3) In contrast to typical ECD, where substrates are coated with FTO/ITO to achieve the required conductivity, we avoided the use of such metal-based current collectors. The coplanar configuration allowed the application of PEDOT-based ink as both the electrochromic dye and current collector. (4) A hydrogel-based electrolyte containing cellulose nanofibers and ILs was incorporated in ECD. (5) While CA has been previously applied as a gel electrolyte in ECDs,^24^ here we report for the first time the use of CAF as a top cover to seal a coplanar ECD.

## Experimental Section

### Materials

CA (CA, Mn ∼ 50,000 g mol^–1^, 39.7 wt % acetyl group, and average degree of substitution, DS = ∼1.2), acetone (>99,5%), 1,2,4,5-benzene tetracarboxylic acid (also known as pyromellitic dianhydride, PMDA, 97%), triethylamine (TEA, ≥99%), tetraethyl orthosilicate (TEOS, 99.99%), octyltrichlorosilane (OCTS, 97%), ethanol (94%, Altia), dimethyl sulfoxide (DMSO, ≥99.9%), and hydrochloric acid (1 M, HCl) were all acquired from Sigma Aldrich. The substrate for the electrochromic displays was a commercial paper (PowerCoat HD230) sized with a PVDC coating and kindly provided by Guarro Casas. The electrochromic ink, PEDOT Orgacon P3165, was provided by AGFA Corporation.

### Cellulose Acetate Films

CAFs were prepared by dissolution of CA (6% and 8% wt %) in acetone under magnetic stirring overnight, followed by casting in a glass Petri dish. The cross-linking reaction was carried out according to our previously reported procedure.^17,18^ Briefly, after CA dissolution, PMDA cross-linker (CA: PMDA molar ratio of 8:1) was added and stirred for 2 h to obtain a homogeneous solution. The stoichiometric CA: PMDA molar ratio for complete cross-linking was 2:1; however, 8:1 CA: PMDA was used to prevent the formation of a rigid cross-linked structure and to ensure that residual hydroxyl groups were available for further modification. To the previous mixture, the TEA (0.5 vol %) catalyst was added slowly and mixed for 1–2 min, and the solution was cast in a glass Petri dish (ø: 13.5 cm) and sealed using a glass lid and Parafilm M. After 48 h, the seals were removed and the system kept in a fume hood to allow for solvent evaporation. All the steps were performed at room temperature.

The effect of CA concentration, cross-linking, and casting volume on the optical and mechanical properties of the films was investigated. In this regard, 12 films were prepared using CA solutions containing 6% and 8% (w/v), as well as 30, 40, and 50 mL cast volumes, with and without cross-linker (Table S1). The preliminary evaluation indicated that the cross-linked CAF obtained from 8% CA and 40 mL volume was most appropriate to produce a film suitable for further surface modification (hydrophobization). This cross-linked CA film is hereafter referred to as CAF-C. A noncross-linked CA film made from 8% CA and 40 mL cast volume (CAF) was used for comparison.

### Film Morphology

Cross sections of the films were analyzed using a scanning electron microscope (LEO-440) with a tungsten filament for generating electrons. The films were fractured after immersion in liquid nitrogen. All the samples were coated with gold. The thickness of the films was obtained from scanning electron microscopy (SEM) images using Image J. Surface roughness of the films was evaluated by atomic force microscopy (AFM) using a MultiMode 8 atomic force microscope from Bruker Corporation (USA). Imaging was carried out via a silicon cantilever (Bruker) in a tapping mode. The processing of the images was performed using NanoScope Analysis 1.5.

### CAF Hydrophobization

The surface of CAF-C (cross-linked films using 8% CA and 40 mL cast volume) was modified using solutions prepared from TEOS and OCTS in ethanol, Milli-Q-water, and HCl. The composition of the sol–gel solution was varied to reach a molar ratio of 0.23:0.13:20:11:0.008 for TEOS/OCTS/EtOH/H_2_O/HCl. A similar methodology was developed by Ding and collaborators using decyltrimethoxysilane.^19^ The solution containing the precursors was stirred for 24 h at room temperature to allow hydrolysis and polycondensation of TEOS and OTCS. Thereafter, CAF-C was immersed in the sol–gel for about 20 s; subsequently, the films were dried under room conditions for 1 h, and then in an oven at 120 °C for 1 h. The CAF-C films that were hydrophobized are referred to as CAF-C-H.

### Film Characterization

Fourier-transform infrared spectroscopy (FTIR) was performed using Bruker, Tensor 27 FT-IR equipment (4000–400 cm^–1^, 32 scans). The transmittance and reflectance of the films were analyzed using an ultraviolet–visible–near infrared Agilent Cary 5000 spectrophotometer in the wavelength range of 200–800 and 250–800 nm, respectively. The thermal stability of the films was evaluated by thermogravimetric analysis (TGA) using a TA Instruments Q500 equipment under a nitrogen atmosphere (10 mL min^–1^) at 10 °C min^–1^ heating rate from room temperature to 800 °C. X-ray diffraction (XRD) analysis was performed using Bruker equipment, model D8 Advance equipped with a LynxEye detector. Measurements were performed from 5 to 60° using 0.025 steps from the initial angle using an irradiation time of 0.5 s per step. The crystallinity indices were acquired using the Ruland method.^25^ This method consists of a simple relationship between the areas of crystalline peaks and the noncrystalline area of X-ray diffractograms. Bruker’s EVA software was used to determine the noncrystalline and crystalline regions of the films.

### Mechanical Strength

Tensile strength and elongation at break of the films were measured using an Instron 4204 Universal Tensile Tester. The films were conditioned for at least 72 h before testing in a controlled relative humidity (RH, 50%) and temperature (25 °C). The specimens were made in rectangular shapes (20 mm × 5.3 mm), with 10 mm grips at both ends. Tensile tests were performed with a 0.5 mm min^–1^ strain rate and repeated at least five times. Dynamic mechanical analyses (DMA) were performed using a TA Instruments DMA 2980. The grips used were of the Tension Film type (length/width) 5.0/5.3 and the testing protocol included 1 Hz frequency, 4 mm amplitude with 0.25 N preload, and 3 °C min^–1^ heating rate from 25 to 260 °C.

### Solvent Resistance

The solvent resistance of the films (CAF, CAF-C, and CAF-C-H) was evaluated by following their solubility and swelling in the respective solvent (water, acetone, and DMSO). The specimens (1 cm^2^) were dried at 105 °C and then immersed in the solvent (5 mL) for 10 min, 20 min, and 24 h under stirring at room temperature. To evaluate the solubility, the undissolved residue was dried and weighted. To determine the swelling degree, the soaked specimens were weighed after the excess of solvent was carefully wiped off with an absorbent paper. The difference between the initial and final weight was used to calculate the % swelling.

### Barrier Properties

Oxygen transmission rate (OTR) through the films was determined according to the ASTM D3985 standard with an Oxygen Permeation Analyzer model 8101 (Systech Instruments Ltd, UK), using at least three replicates. The tests were carried out at 23 °C and 80% RH using metal masks (5 cm^2^ test area) in 100% oxygen. The humidity gradient was used as a driving force for water molecules to diffuse within the films. Thus, water vapor transmission rates (WVTR) were measured gravimetrically using a modified ASTM-E-96 B procedure “dry cup method”. Samples with a test area of 30 cm^2^ were mounted on circular aluminum cups (68-3000 Vapometer EZ-Cups; Thwing-Albert Instrument Company) containing anhydrous CaCl_2_ (0% RH). The cups were stored under the test conditions (23 °C and 50% RH) and weighed periodically until attaining a constant weight reduction. A Climaveneta climate control system (model AXO 10, Italy) was used to control both temperature and humidity in an environmental room. The tests were repeated four times at a 50 to 0% RH gradient. The water contact angle was evaluated using a Contact Angle and Surface Tension Meter, CAM200 (KSV Instruments Ltd., Finland). 2 μL of water was placed on the films, and the contact angle was measured after 10 min under ambient conditions of controlled temperature (25 °C) and RH (50%).

### Electrochromic Displays

**Figure 1:**
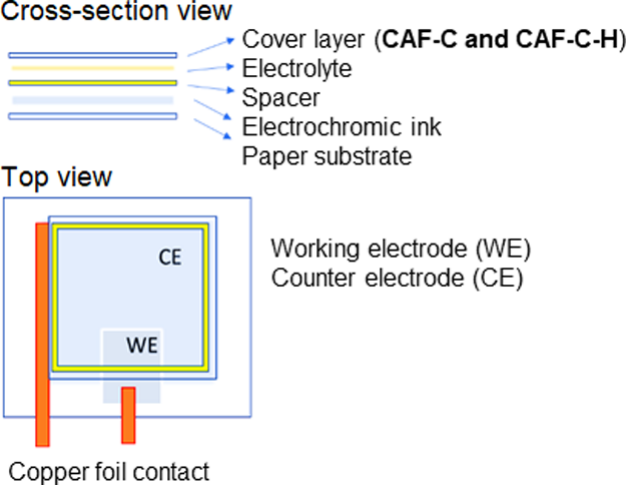
Schematic illustration of the coplanar ECD based on a paper substrate and CAF used as cover layers.

Paper-based electrochromic displays (ECDs) were fabricated using PowerCoat HD230 (paper) as substrates. Additionally, PowerCoat papers were coated with PVDC to improve their barrier features. CAF-C and CAF-C-H were applied as a top layer to laminate the electrochromic (EC) dye (PEDOT Orgacon P3165) and the electrolyte. A new type of hydrogel-based electrolyte was applied, composed of an ionic liquid (butyl-1-methylpyrrolidinium trifluoromethanesulfonate) and TEMPO-oxidized cellulose nanofibrils (TOCNF) (50:50). ECDs were prepared in a coplanar architecture, shown in Figure 1.

The paper substrate was coated with EC ink by screen printing (screen-printer Etiem Lab 1000, 3.5 bar, 6 mm snap off, and 35 mm elevation at 250/300 mm s^–1^ on a 110/40Yscreen). After printing, the substrates were dried for 1.5 min at 130 °C to cure the ink. Then, a spacer (50 μm thickness) was added using double-face tape to define the pool of electrolyte, followed by the electrolyte deposition, which was manually applied. Copper foils were fixed on the EC ink to provide contacts between the counter and working electrodes, respectively. Finally, CAF-C and CAF-C-H were laminated as a cover layer.

## Results and Discussion

### Cross-Linking and Hydrophobization

**Figure 2:**
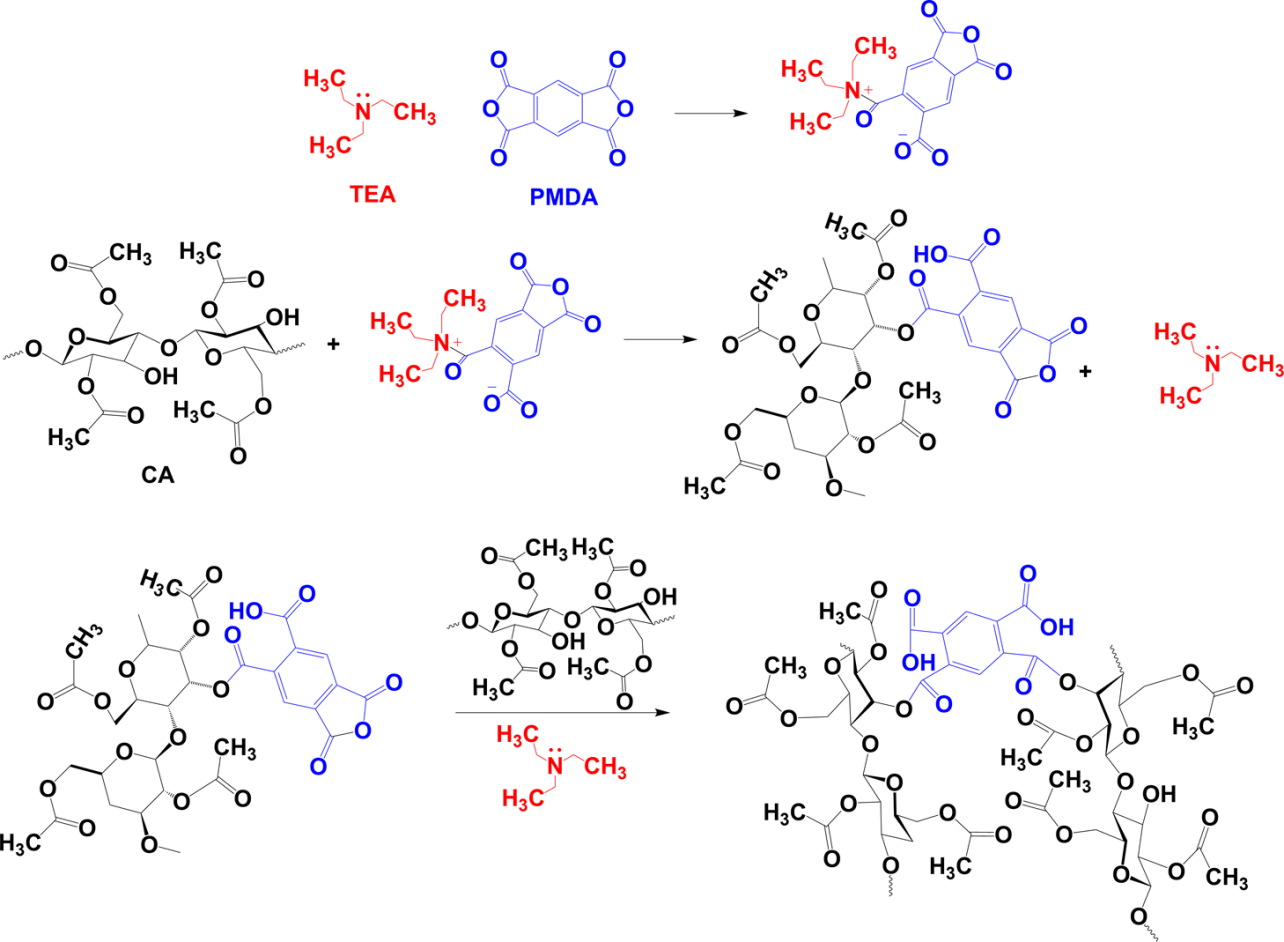
Proposed reaction mechanism for cross-linking of CA using PMDA and TEA.

Both cross-linked and noncross-linked CAF were prepared using two different solutions containing 6 and 8% of CA. The volume of cast solutions was also varied (30, 40, and 50 mL) to investigate the effect of the thickness on CA film properties. The cross-linked films were prepared using PMDA as a cross-linker and TEA as a catalyst (Figure 2). The most likely mechanism in the cross-linking reaction includes the attack of the nonbonding nitrogen pair of electrons of TEA to the PMDA carbonyl group and the formation of an activated intermediate. Next, the free hydroxyl groups of CA attack the carbonyl carbon of the activated intermediate, resulting in the release of the catalyst back to the reaction medium, followed by a proton transfer and product formation. This product reacts with another CA molecule, following the same mechanism, which leads to cross-linking between adjacent CA chains.

The FTIR spectra of the cross-linked films (Figure S1) show low-intensity bands at approximately 1500 and between 690 and 900 cm^–1^, which are attributed to the absorption of aromatic rings introduced by the cross-linker. Compared to the noncross-linked films, the increased number of ester groups, because of the cross-linking between CA chains, is evidenced by the increased intensity of the C=O stretch band (∼1730 cm^–1^).

**Figure 3:**
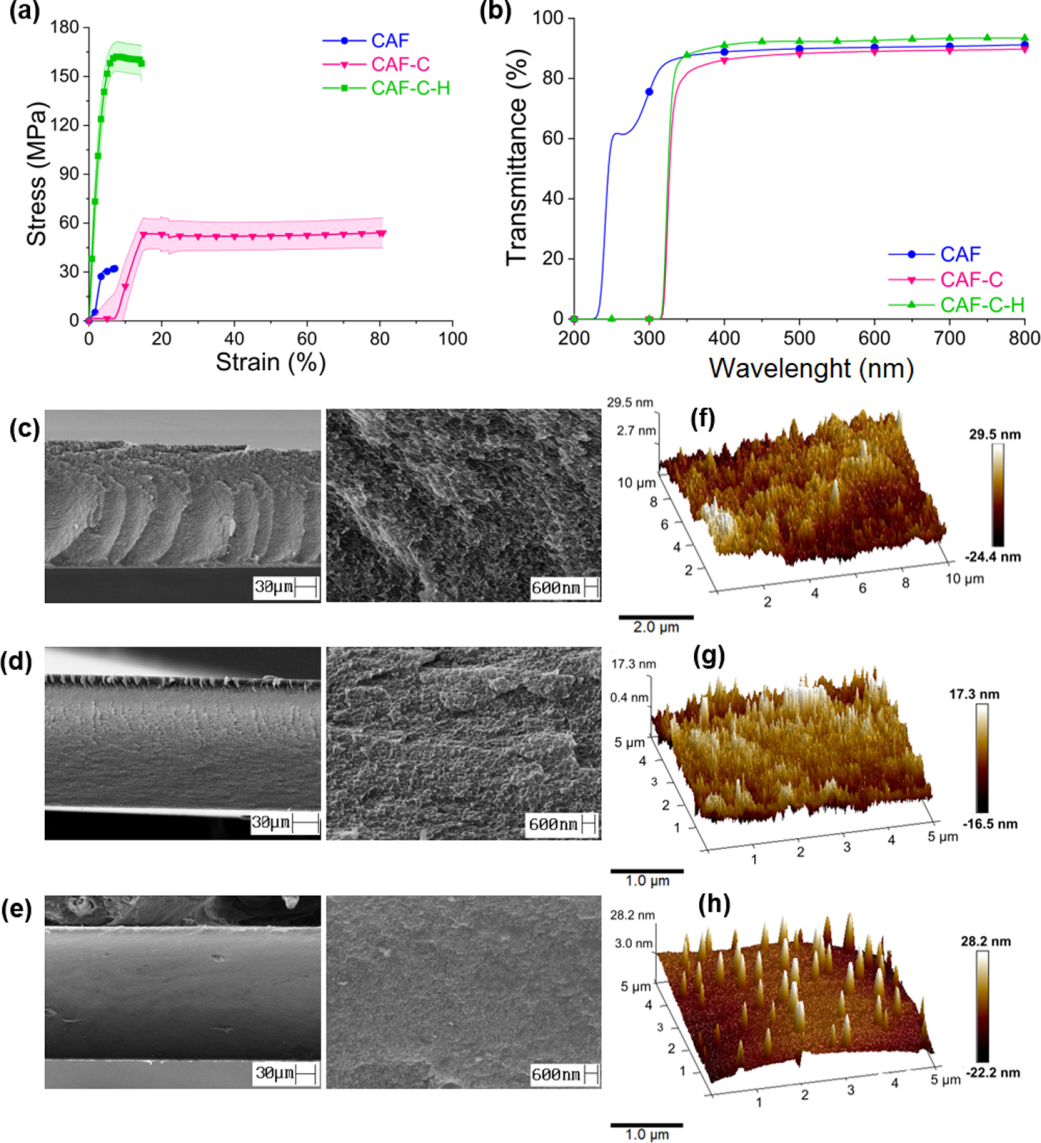
(a) Tensile curves, (b) UV–vis spectra, (c–e) SEM micrographs (from cross sections), and (f–h) AFM images (from surface) of CAF, CAF-C, and CAF-C-H.

The tensile properties of all the 12 films are presented in Table S1 and Figure S2. Overall, cross-linking of CA provided the films with better tensile strength and increased elongation at break, for example, better toughness. The effect of cross-linking on tensile properties was more evident in the films obtained with 8% CA solution, which presented increased tensile stress, from 32.0 ± 0.6 MPa (noncross-linked film) to 67.0 ± 2.0 MPa (cross-linked film). The largest impact of cross-linking on the elongation at break corresponded to the film obtained with 8% CA solution and 40 mL casting volume, reaching 81% strain compared to the noncross-linked film (only 7%) (Figure 3a). The cross-linked films produced from the 8% CA solution and 40 mL casting volume (labeled as CAF-C) presented the best mechanical performance and were considered for further hydrophobization and application in electrochromic displays.

Hydrophobization was performed on CAF-C via sol–gel processing through the reaction between the precursors and the free surface hydroxyls (Figure S3). First, TEOS was hydrolyzed to form silicic acid, followed by alcoholic and aqueous condensation, which led to the formation of a polysiloxane network (gel). This gel, rich in hydroxyl groups, reacted with the remaining hydroxyl groups of CAF-C, then covalently linking the polysiloxane to the film. Similarly, OCTS underwent substitution followed by condensation leading to the formation of long alkyl chains that interacted with the hydroxyls of the gel, thereby increasing the number of hydrophobic groups on the surface.^19,31^ The presence of silicon groups on CAF-C-H films was confirmed by FTIR (Figure S4), with bands at 1115 cm^–1^ (symmetric and asymmetric stretching vibrations of −Si–O–Si−) and low-intensity bands between 2850 and 2955 cm^–1^ (related to the stretching vibration of n-Si and p-Si).

The effect of cross-linking and hydrophobization on tensile properties of the films is shown in Figure 3a and Table 1. The cross-linking reactions increased the packing density of CAF-C compared to CA, resulting in a significant increase in the mechanical performance (tensile strength from 32 to 54 MPa, Young modulus from 1 to 1.9 MPa, and elongation at break from 7.3 to 80.5%). Hydrophobization made significantly more rigid films (CAF-C-H with Young’s modulus increased to 4.5 MPa), more resistant to traction (tensile strength of 139 MPa), but less extensible (strain at break of ∼13.5%).

**Table 1:** Thickness, Crystallinity Index, Reflectance, and Transmittance (at 550 nm) as well as Tensile Properties for CAF, CAF-C, and CAF-C-H

	CAF	CAF-C	CAF-C-H
thickness (μm)	178 ± 7	157 ± 4	207 ± 8
crystallinity index (%)	23.0 ± 0.5	17.0 ± 0.5	17.0 ± 0.5
reflectance at 550 nm (%)	7.2	7.7	4.0
transmittance at 550 nm (%)	90	87	92
Young Modulus (MPa)	1.00 ± 0.09	1.9 ± 0.2	4.4 ± 0.4
tensile stress at break (MPa)	32.0 ± 0.6	54.0 ± 8.0	139.0 ± 10.4
tensile strain at break (%)	7.3 ± 1.2	80.5 ± 9.1	13.5 ± 1.9

### Morphology and Optical Properties

CAF presented surfaces dominated by ripple-like structures (Figure S5), probably generated by the rapid evaporation of the solvent (acetone), as also observed by Gomez-Hermoso-de-Mendoza and collaborators (2020).^26^ The precursor of CAF-C produced a cross-linked gel, which slowed down solvent evaporation, leading to a homogeneous surface. With the increase of the CA concentration (from 6 to 8%) and casting volume (from 30 to 50 mL), the thickness of the films was increased. Meanwhile, no significant impact was observed on their optical properties (transmittance and reflectance at 550 nm, Figure S6 and Table S1). After hydrophobization, there were no significant changes in the appearance of CAF-C-H compared to that of CAF-C.

The UV–Vis spectra of CAF, CAF-C, and CAF-C-H are shown in Figure 3b. All the films exhibited transparency >87%. Note that the transparency of CAF depends highly on the degree of acetylation and molecular weight, and our results compared favorably with those reported earlier (80%).^26,27^ Cross-linking and surface modifications did not significantly affect the transparency of the films (at 550 nm). However, the transmittance under 325 nm was significantly altered by cross-linking, and the peak near 250 nm was not detected, because of the elimination of aldehyde groups.^28−30^ A slight increase in transmittance, as well as a decrease in reflectance (around 4% at 550 nm), was observed for CA-C-H, which was likely because of the additional silanol groups and the denser film structure (Figure 3b).

In addition to the changes in the chemical structure, cross-linking and hydrophobization treatment affected the film thickness (Table 1) and morphology (Figure 3). Cross-linking reduced the film thickness, from 178 ± 7 μm (CAF) to 157 ± 4 μm (CAF-C), which was attributed to a better packing or cohesion within the film structure. The surface modification (hydrophobization) increased the thickness to 207 ± 8 μm for CAF-C-H. Cross-linking between CA chains made it difficult to align neighboring segments, and thus the crystallinity decreased from 23% (CAF) to 17% for the cross-linked film (CAF-C). In this case, the crystalline domains were probably generated by the alignment of longer segments not involved in cross-linking (diffractograms not shown). After hydrophobization, the crystallinity remained at 17%, confirming that the chemical reaction took place on the surface and did not affect the bulk of the film (Table 1).

SEM images of the cross section of the films (Figure 3c–e) show that CAF-C was denser than CAF, as expected because of the cross-linking of the CA segments. CAF-C-H films were even denser than those from CAF-C, because of the sol–gel process and the soaking of the film in the solvent (containing ethanol/water/acid) followed by oven-drying, which resulted in further packing of the CA segments. The surface morphology observed by AFM showed a smoother surface after cross-linking (Figure 3f–h). Considering the scale bars, the surface roughness in CAF was almost 54 nm compared to 34 nm for CAF-C, confirming the absence of rippling structures at the microscopic scale. The hydrophobization led to silicon nanostructures (spikes) on the surface, with a height up to 28 nm.

### Thermal Properties

**Figure 4:**
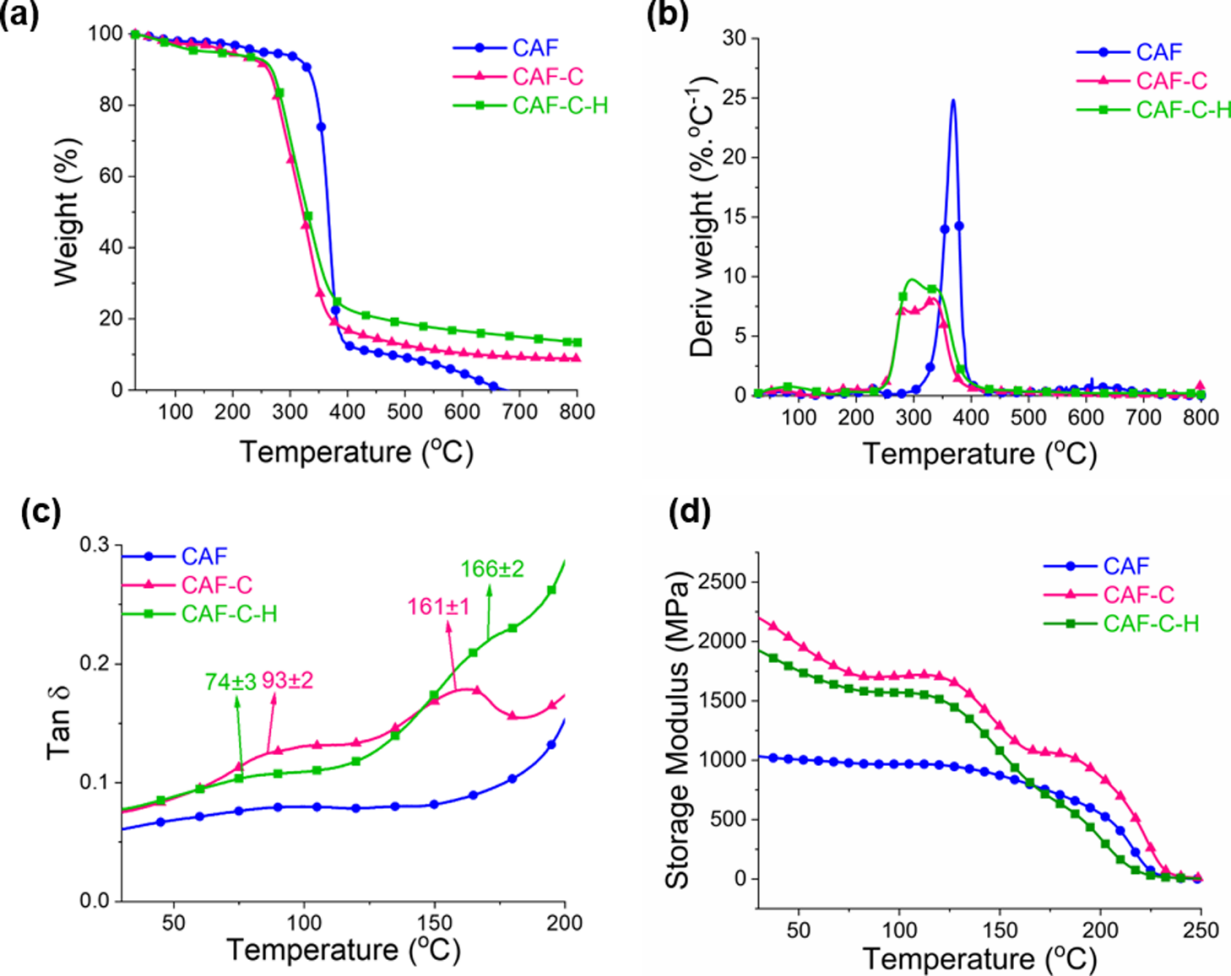
(a) TG and (b) DTG profiles (N_2_ flow 10 mL min^–1^ and 10 °C min^–1^ heating rate), (c) Tan δ and (d) storage modulus curves of CAF, CAF-C, and CAF-C-H.

The effect of cross-linking and hydrophobization on the thermal properties of the films was also investigated (Figure 4a,b). The thermal decomposition of CA included several reactions, such as deacetylation, scission of cellulosic chains, and liberation of compounds such as acetic acid, H_2_O, CO_2_, acetyl derivatives, and furans.^32,33^ The thermal event observed in the TGA profile of CAF, and the peak related to the maximum decomposition ratio (368 °C, DTG curve, Figure 4b) can be attributed to the thermal decomposition typical of CA, as mentioned above. The DTG curve of CAF-C exhibited two peaks, one at 282 °C, which may be assigned to the thermal decomposition involving the cross-linking bonds (which shifted the T-onset from 340 to 266 °C), and the other at 335 °C, related to the decomposition of the CA backbone. The DTG profile for CAF-C-H also showed two peaks, similar to those of CAF-C (Figure 4b). The residual mass increased from CAF (1%) to CAF-C (9%) and CAF-C-H (14%), as expected. This observation is explained by the moieties added upon cross-linking (CAF-C) and subsequent hydrophobization (CAF-C-H), respectively. This serves as further confirmation of successful cross-linking and hydrophobization of CAF-C films.

DMA analyses were used to determine the glass transition and storage modulus of the films (Figure 4c,d). The glass transition of CA is related to the rotational motions involving the single covalent bonds present in the segments of the noncrystalline domains.^9^ Cyclic structures, like those present in CA, restrict these movements, which is likely to decrease the number of rotating chemical bonds. The increase in the storage modulus (mainly from 25 °C to approximately 125 °C), from CAF to CAF-C, can be attributed to the cross-linking between the CA chains, which led to a stiffer film. No significant difference was observed between the storage moduli values of CAF-C and CAF-C-H (Figure 4d). Tan δ curve of CAF showed a peak at approximately 92 °C, which was attributed to its *T*_g_. The *T*_g_ values of CAs depend on their average molar mass, the average degree of substitution, and on the physical state/technique through which they are analyzed (e.g., pulverized, in analysis via differential scanning calorimetry, DSC, or as films, via DMA). No report was found in the literature for the *T*_g_ values of films with characteristics similar to those of this study, which prevented a comparative analysis. In the Tan δ curves of CAF-C and CAF-C-H, the peaks were observed at approximately 93 °C and 74 °C, respectively, which can be attributed to *T*_g_ of long segments not involved in cross-linking. The peaks observed at higher temperatures (161 and 166 °C, respectively, for CAF-C and CAF-C-H) can be attributed to *T*_g_ related to shorter segments between cross-links.

### Barrier Properties and Solvent Resistance

**Figure 5:**
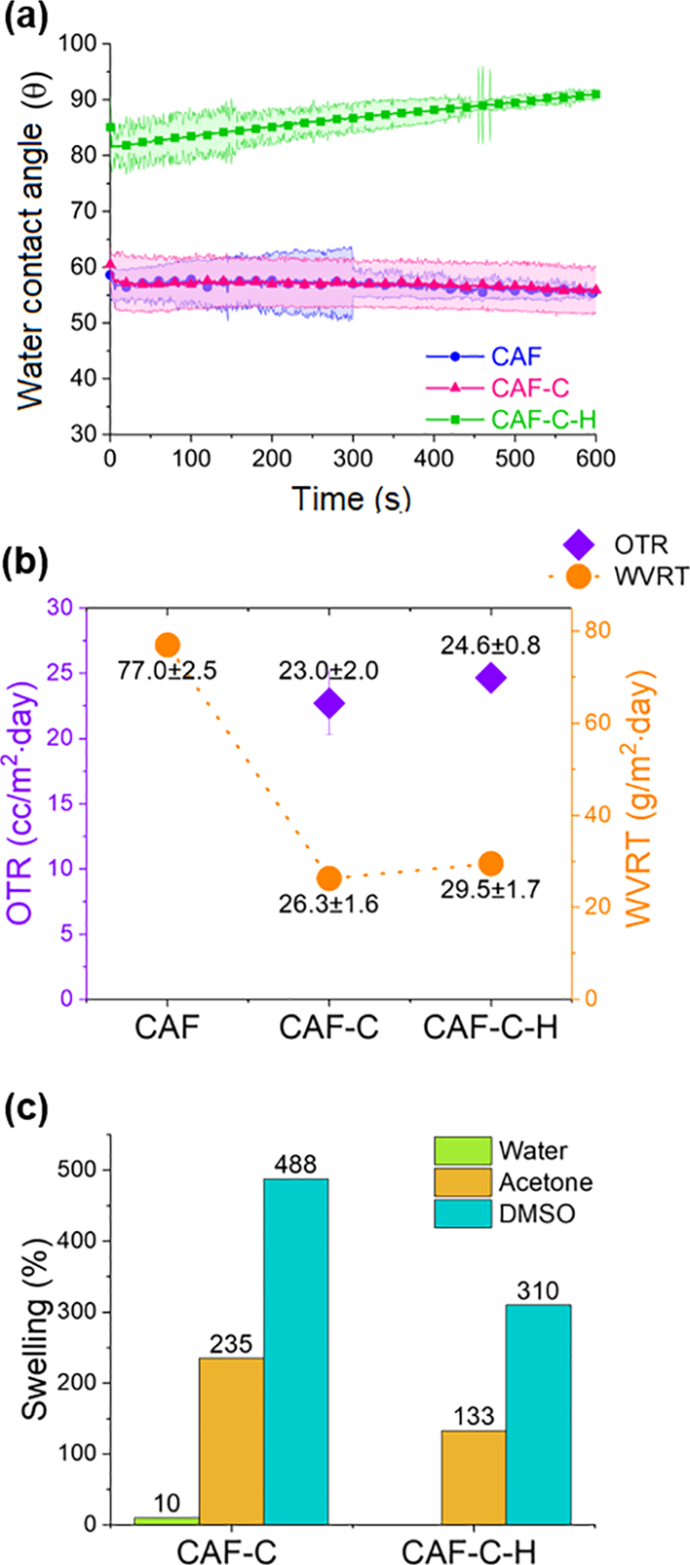
(a) Water contact angle, (b) oxygen and water vapor transmission rate, and (c) swelling rate of CAF, CAF-C, and CAF-C-H in water, acetone, and DMSO after 24 h.

Figure 5a shows that cross-linking did not contribute to the hydrophobicity of the films: the water contact angle remained fairly similar, 55.3 ± 0.7° (CAF) and 56.0 ± 4.2° (CAF-C). Hydrophobization reduced water droplets’ interaction with the CAF-C-H surface, leading to a higher water contact angle (91.0 ± 1.1°). Meanwhile, cross-linking significantly improved the moisture barrier, as seen from water vapor transfer rates (WVTR) in Figure 5b: 77.0 g/m^2^ day (CAF) to 26.3 g/m^2^ day (CAF-C). After hydrophobization of the cross-linked film, the moisture barrier was slightly decreased (29.5 g/m^2^ day).

The barrier to oxygen transfer was analyzed by OTR (Figure 5b). OTR was not measurable for the uncross-linked film (CAF) because of its wrinkly pattern, which could not be kept hold during the experiment. However, cross-linking significantly decreased OTR (CAF-C: 22.7 cc/m^2^ day) almost over 10 times lower than OTR values reported for CAF in the literature (280 cc/m^2^ day).^34^ Like the moisture barrier, hydrophobization treatment slightly decreased the oxygen barrier reaching 25.0 ± 0.8 cc/m^2^ day for CAF-C-H.

The films’ dissolution resistance in common organic solvents, including acetone, DMSO, and water was investigated (Figure 5c). CAF was completely dissolved in acetone and DMSO in 20 min while showing 3% weight losses in water. After the cross-linking, the CAF-C became insoluble in water. The CAF-C film swelled (10% increase) in water, whereas it dissolved (18%) in acetone after 24 h, indicating that a small fraction of polymer was not cross-linked. DMSO did not dissolve CAF-C, rather the film swelled more extensively compared to that in acetone, likely because of the higher polarity of DMSO. Although cross-linking of CAF significantly improved solvent resistance, swelling of the films in the presence of organic solvents may limit their applications. Successfully, hydrophobization treatment decreased the swelling by 43 and 37% for acetone and DMSO, respectively. The hydrophobic groups on the surface protected the films from interacting with the organic polar solvents and prevented swelling or further solvent penetration in the structure.

### Paper-Based Electrochromic Display

CAF-C and CAF-C-H were applied as a top cover in a flexible ECD designed with a coplanar architecture. The cell included a paper substrate, printed PEDOT-based dye, electrolyte, and a top cover to seal and avoid evaporation of the electrolyte (Figure 1). The PVDC coating on the paper substrate provided an extra barrier to the electrolyte hydrogel. The durability over time was monitored by activating the ECD every 30 min and applying −3 V to the working electrode (WE) (dark blue color) followed by deactivation at 0 V or +3 V (light blue color). The switching voltage of ±3 V was chosen given that in a coplanar configuration the color change occurs slower than when in a sandwiched configuration; moreover, we found the optimum voltage to be 2.9 V. Upon applying the potential, the EC ink (PEDOT) changes to dark blue when it is reduced (−3 V). When switching off the display (WE), in the neutral state (0 V) or the oxidized state (+3 V), the EC ink changes to a light blue. The display was activated until no color change was visually observed.

**Figure 6:**
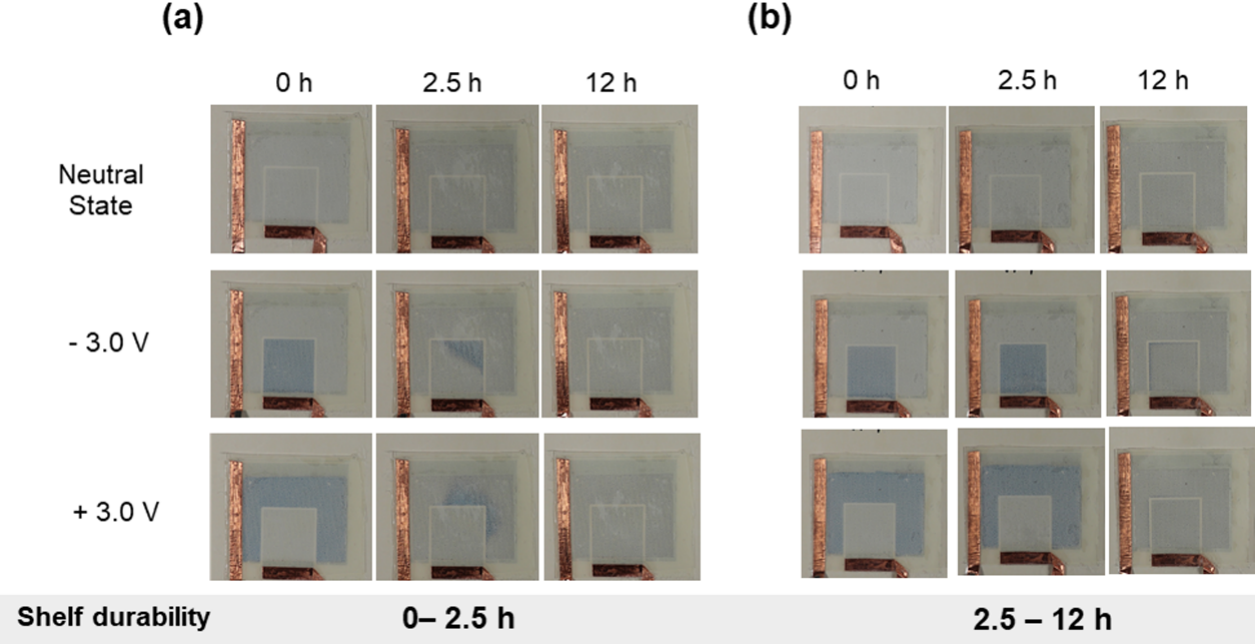
Durability of coplanar ECDs prepared on a paper substrate and using a cove film of (a) CAF-C and (b) CAF-C-H.

As can be seen in Figure 6, when using the CAF-C as a cover layer, the color in the ECD faded after 2.5 h at −3, 0, or +3 V. In contrast, for CAF-C-H, the color continued cycling for at least 12 h. The obtained results show that compared to those with CAF-C, the coplanar ECDs based on CAF-C-H retained their functionality for a longer period of time. Meanwhile, the ECD’s half-a-day performance can be explained by the oxidation and drying of the electrolyte. It was noted that the electrolyte containing TOCNF and IL was prone to fast evaporation, and most of the water was already evaporated during the cell assembly. Besides, the electrolyte was prone to oxidation given that a brownish color developed during one-week preservation. Further work is needed to optimize the electrolyte composition and the performance of the ECD. Nevertheless, our observations indicate that the hydrophobization of cross-linked CA film improved the barrier properties, providing better isolation and sealing of the device, increasing the shelf life and functionality of the assembled displays.

## Conclusions

Cellulsoe acetate films (CAFs) with modified microstructure and surface properties were obtained by cross-linking and hydrophobization treatments. Cross-linking was carried out using PMDA as a cross-linker and TEA as a catalyst. Hydrophobization was applied on the cross-linked film via sol–gel method containing TEOS and OCTS. Cross-linking reaction formed a cross-linked gel, which slowed down solvent evaporation after casting the solution, leading to a more homogeneous film without wrinkles after drying. Besides, cross-linking increased the packing of the CA chains resulting in a significantly improved barrier to oxygen and moisture (from 77.0 to 26.3 g/m^2^ day) and solvent resistivity (acetone and DMSO), as well as mechanical properties (tensile strength from 32 to 54 MPa, Young modulus from 1 to 1.9 MPa, and elongation at break from 7.3 to 80.5%), Upon hydrophobization, the films become denser, rigid, and less extensible (Young’s modulus increased to 4.5 MPa, tensile strength to 139 MPa, and strain at break of ∼13.5%). While hydrophobization treatment increased the contact angle (from ∼56 to 91°) and solvent resistivity, there was no significant effect on barrier properties. When applied as the top cover in a coplanar ECD, the film obtained by cross-linking and hydrophobization extended the functionality of the display compared to the cross-linked film. The results indicate excellent prospects for CAF in achieving more environmental-friendly ECDs to replace PET-based counterparts.

## Supporting Information Available

Thickness, transmittance, reflectance, young modulus, tensile stress, and tensile strain from noncross-linked and cross-linked CAF, the respective concentration of CA (6% and 8%), and volume of solution (30, 40, and 50 mL); FTIR spectra of the noncross-linked films prepared from CA solutions with concentrations of 6 and 8%, and the cross-linked films using 6 and 8%, and the solution volumes; tensile analysis for the noncross-linked films 6 and 8% and the cross-linked films 6 and 8%; UV–vis analysis of the noncross-linked films prepared from CA solutions with concentrations of 6 and 8%, and the cross-linked films using 6 and 8%, and the solution volumes; hydrophobization of cross-linked CAFs via the sol–gel process using TEOS and OTCS; and FTIR for CAF-C and CAF-C-H (PDF)

## Abbreviations

CA: cellulose acetate
CAF: noncross-linked cellulose acetate film
CAF-C: cross-linked cellulose acetate film
CAF-C-H: cross-linked and hydrophobized cellulose acetate film
PMDA: pyromellitic dianhydride
TEOS: tetraethyl orthosilicate
OCTS: octyltrichlorosilane
ECD: electrochromic devices
DMSO: dimethyl sulfoxide
AFM: atomic force microscopy
FTIR: Fourier-transform infrared spectroscopy
XRD: X-ray diffraction
RH: relative humidity
DMA: dynamic mechanical analyses
OTR: oxygen transmission rate
WVTR: water vapor transmission rates
