# Supplementary material for: Cross-Linked and Surface-Modified Cellulose Acetate as a Cover Layer for Paper-Based Electrochromic Devices
Source: ACS Appl Polym Mater. 2021 Mar 16;3(5):2393–401. doi: 10.1021/acsapm.0c01252 (PMC8290922; doi:10.1021/acsapm.0c01252)
Supplement: Supplementary file 1 — ap0c01252_si_001.pdf [file ap0c01252_si_001.pdf]

SUPPORTING INFORMATION

Cross-linked and surface-modified cellulose acetate as a cover layer for paper-based electrochromic devices

*Joice Jaqueline Kaschuk[1,2], Maryam Borghei[2], Katariina Solin[2], Anurodh Tripathi[2,3], Alexey Khakalo[4], Fábio A. S. Leite[5], Aida Branco[6], Miriam C. Amores de Sousa[5], Elisabete Frollini[1], Orlando J. Rojas[2,7]**

[1]Macromolecular Materials and Lignocellulosic Fibers Group, Center for Research on Science and Technology of BioResources, Institute of Chemistry of São Carlos, University of São Paulo, CP 780, 13560-970 São Carlos, São Paulo, Brazil

[2] Department of Bioproducts and Biosystems, School of Chemical Engineering, Aalto University, Vuorimiehentie 1, FI-00076, Espoo – Finland

[3] Department of Chemical and Biomolecular Engineering, North Carolina State University, Raleigh, NC 27695, United States

[4] VTT Technical Research Centre of Finland Ltd, P.O. Box 1000, FI-02044, VTT, Finland

5. Ynvisible GmbH, Engesserstr. 4A 79108 Freiburg, Germany

[6] Ynvisible SA, Rua Quinta do Bom Retiro 12C, 2820-690, Charneca da Caparica, Portugal

[7] Bioproducts Institute, Department of Chemical and Biological Engineering, Department of Chemistry and Department of Wood Science, The University of British Columbia, 2360 East Mall, Vancouver, BC V6T 1Z3, Canada

*Corresponding author: Orlando J. Rojas: orlando.rojas@ubc.ca

Table S1 – Thickness, Transmittance, Reflectance, Young Modulus, Tensile Stress and Tensile strain from non-crosslinked and crosslinked cellulose acetate films, the respective concentration of cellulose acetate (6%, 8%), and volume of solution (30mL, 40mL, 50mL)

	**Non-crosslinked**						**Crosslinked**					
	**6%**			**8%**			**6%**			**8%**		
	**30 mL**	**40 mL**	**50 mL**	**30 mL**	**40 mL**	**50 mL**	**30 mL**	**40 mL**	**50 mL**	**30 mL**	**40 mL**	**50 mL**
**Tickness (µm)**	99±9	112±8	121±29	116±6	178±7	243±16	85±4	109±14	104±4	96±1	157±4	206±8
**Transmittance (%)**	88	86	84	88	90	83	90	90	89	91	87	87
**Reflectance (%)**	7.4	7.7	7.8	7.5	7.2	6.2	7.5	6.4	7.6	7.4	7.7	7.8
**Young Modulus (MPa)**	1.6±0.2	1.3±0.03	1.2±0.2	1.4±0.2	1.00±0.09	1.8±0.4	1.5±0.2	1.6±0.3	1.2±0.1	1.4±0.01	1.9±0.2	1.9±0.2
**Tensile Stress at break (MPa)**	48.0±0.2	41.0±0.1	36.0±0.6	41.0±0.4	32.0±0.6	35.0±0.7	54.0±0.7	59.0±1.3	51.0±0.5	64.0±4.0	54.0±8.0	67.0±2.0
**Tensile Strain at break (%)**	8.2±1.8	17.2±1.0	9.4±2.7	8.5±0.7	7.3±1.2	7.7±0.1	47.4±6.5	55.1±7.4	74.0±8.0	51.1±3.3	80.5±9.1	69.4±0.9

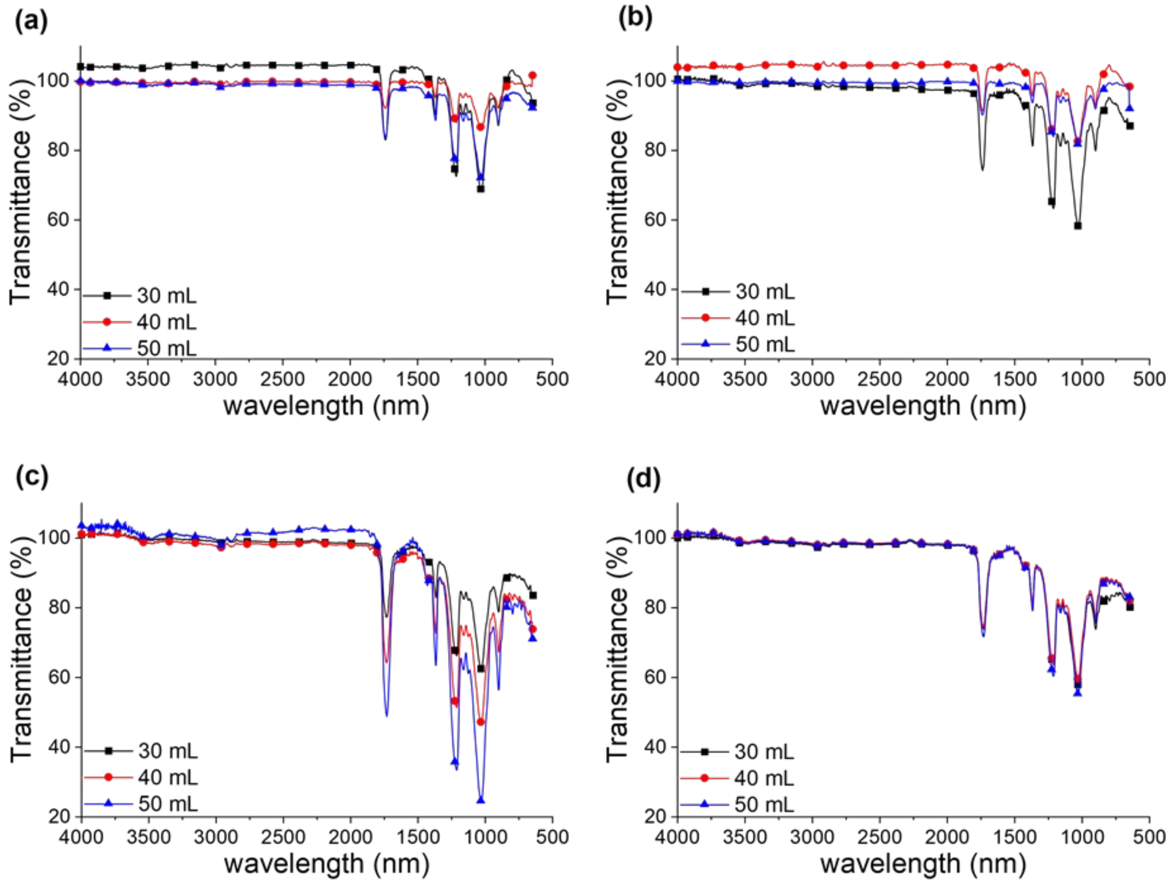

Figure S1 - FTIR spectra of the non-crosslinked films prepared from CA solutions with concentrations of 6% (a) and 8% (b), and of the crosslinked films using 6% (c) and 8% (d), and the solution volumes shown from (a) to (d)

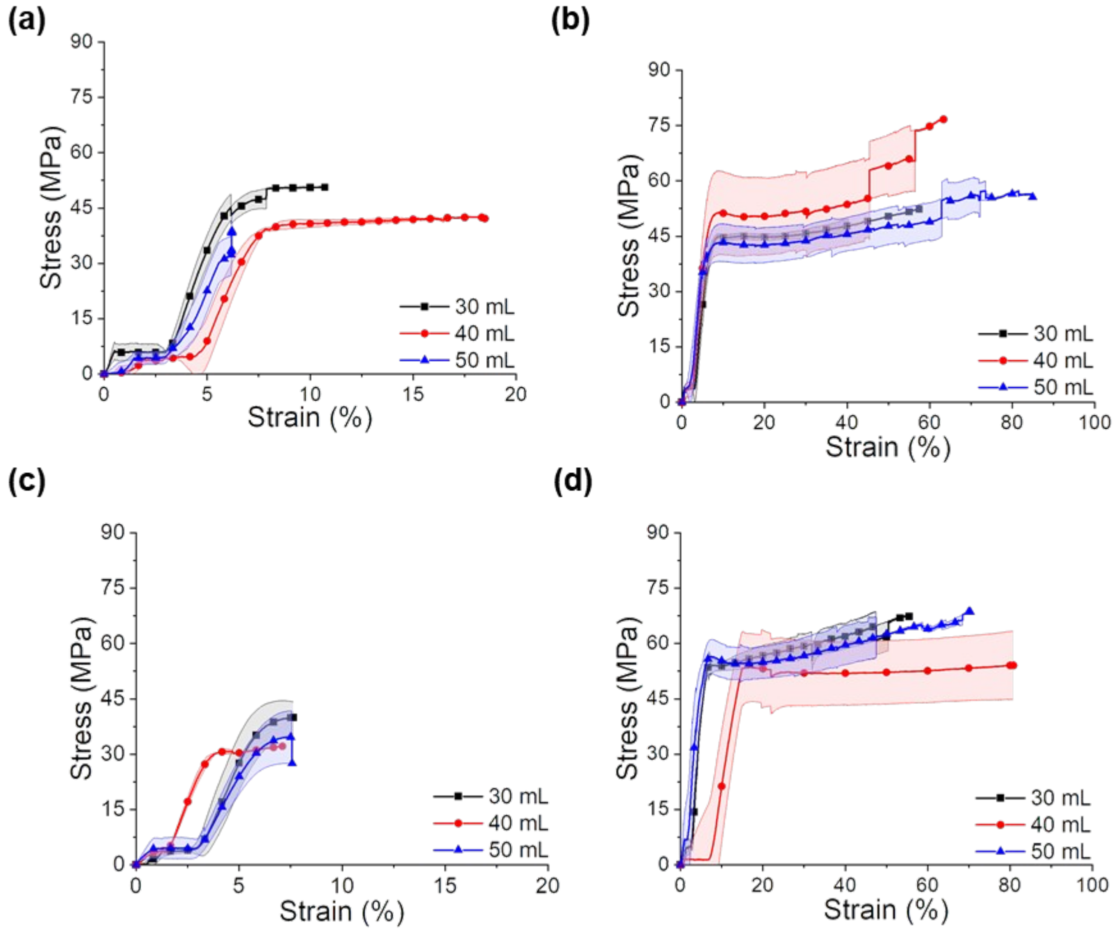

Figure S2- Tensile analysis for the non-crosslinked films (a) 6% and (c) 8%, and the crosslinked films (b) 6% and (d) 8%.

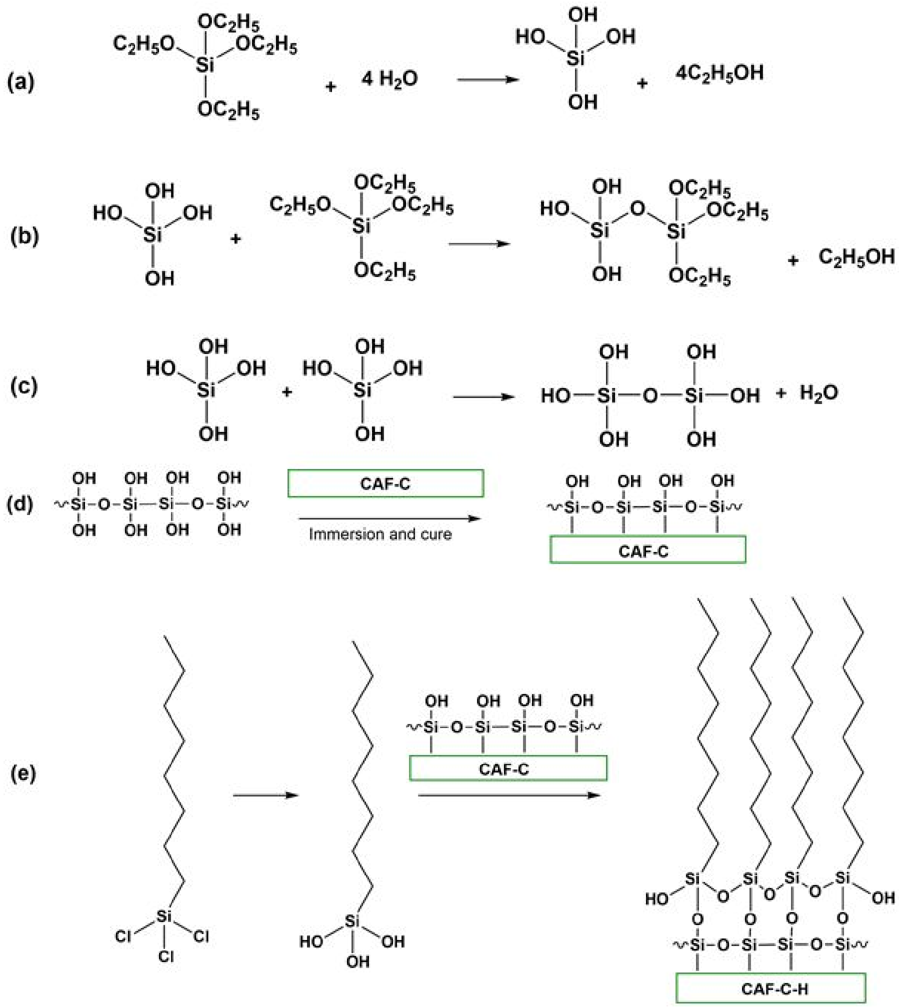

Figure S3 - Hydrophobization of crosslinked CA films via sol-gel process using TEOS and OTCS.

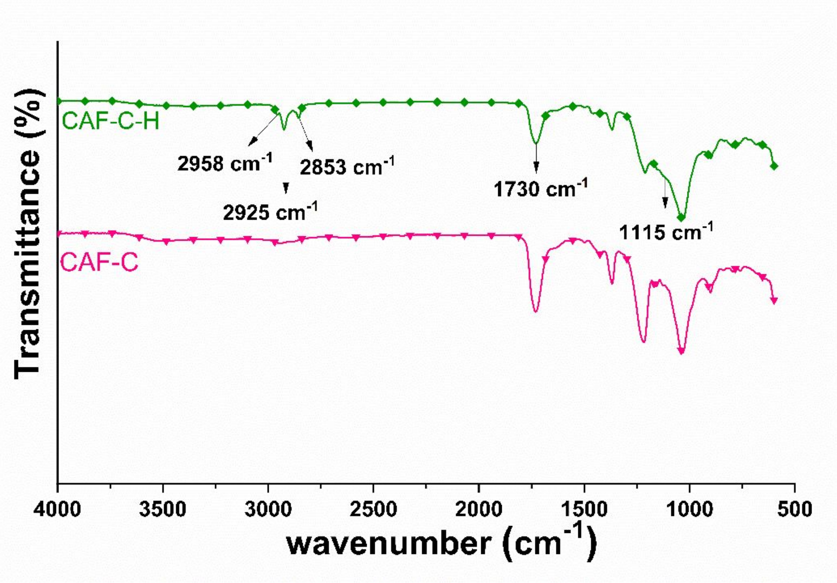

Figure S4- FTIR for CAF-C and CAF-C-H.

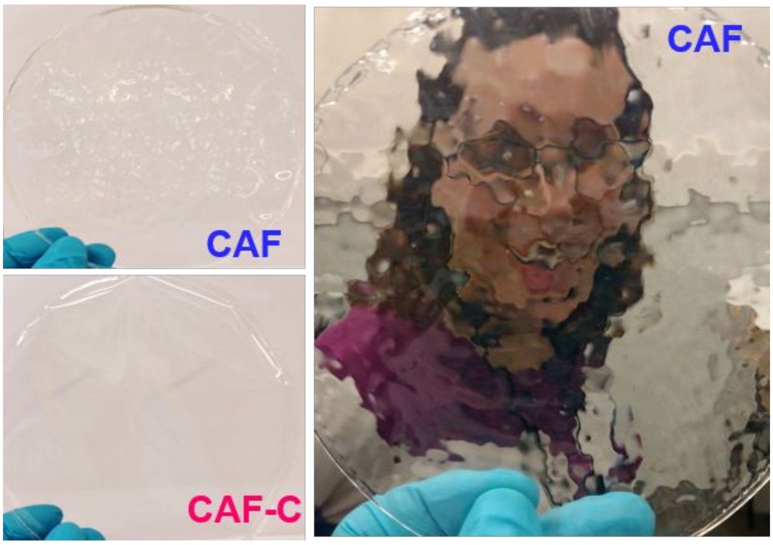

Figure S5. Digital photos of non-crosslinked (CAF) and crosslinked CA films (CAF-C).

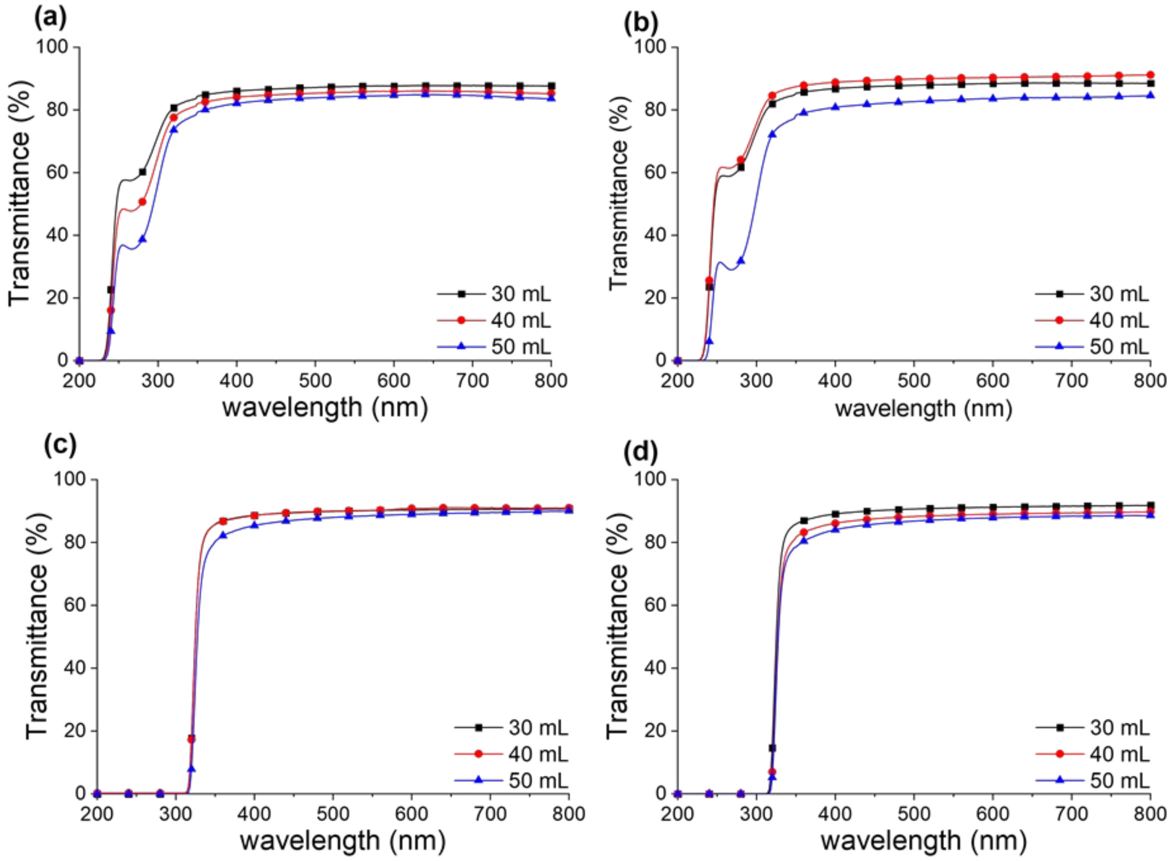

Figure S6- UV-vis analysis of the non-crosslinked films prepared from CA solutions with concentrations of 6% (a) and 8% (b), and of the crosslinked films using 6% (c) and 8% (d), and the solution volumes shown from (a) to (d)
